# Morpho-Phylogenetic Evidence Reveals Novel Species and New Records of *Botryosphaeriaceae* in China and Thailand

**DOI:** 10.3390/jof9111051

**Published:** 2023-10-26

**Authors:** Na Wu, Asha J. Dissanayake, Hong-Zhi Du, Jian-Kui Liu

**Affiliations:** 1 School of Life Science and Technology, Center for Informational Biology, University of Electronic Science and Technology of China, Chengdu 611731, China; wuna220@gmail.com (N.W.); asha.janadaree@yahoo.com (A.J.D.); hongzhi_du1012cc@163.com (H.-Z.D.); 2 Center of Excellence in Fungal Research, School of Science, Mae Fah Luang University, Chiang Rai 57100, Thailand; 3 School of Pharmacy, Guizhou University of Traditional Chinese Medicine, Guiyang 550025, China; 4 Department of Entomology and Plant Pathology, Faculty of Agriculture, Chiang Mai University, Chiang Mai 50200, Thailand

**Keywords:** 4 new taxa, asexual morph, multi-gene, phylogeny, sexual morph, taxonomy

## Abstract

Species in the *Botryosphaeriaceae* are common plant pathogens, endophytes, and saprobes found on a variety of mainly woody hosts. *Botryosphaeriaceae* is a high-profile fungal family whose genera have been subjected to continuous revisions in recent years. Surveys conducted during 2019 and 2020 on several decaying woody hosts (from dead arial twigs, branches, stems, bark, and seed pods) in China and Thailand revealed a high diversity of *Botryosphaeriaceae* fungi. Identification of 16 *Botryosphaeriaceae* isolates was carried out based on both morphological characteristics and phylogenetic analyses of combined ITS, LSU, *tef1-α*, and *tub2* sequence data. Four novel species (*Dothiorella ovata*, *Do. rosacearum*, *Do. septata*, and *Lasiodiplodia delonicis*) and seven previously known species (*Botryosphaeria fujianensis*, *Diplodia mutila*, *Di. seriata*, *L. crassispora*, *L. mahajangana*, *Macrophomina euphorbiicola* and *Sphaeropsis eucalypticola*) were identified while new hosts and geographical records were reported. This study indicates that the fungal family *Botryosphaeriaceae* seems to be common and widespread on a broad range of hosts in China and Thailand.

## 1. Introduction

The order *Botryosphaeriales* (Dothideomycetes) was established by Schoch et al. [1], including a single family *Botryosphaeriaceae*. A recent comprehensive study by Phillips et al. [2] accepted six families, viz. *Aplosporellaceae*, *Botryosphaeriaceae*, *Melanopsaceae*, *Phyllostictaceae*, *Planistromellaceae*, and *Saccharataceae* are in this order based on morphological and phylogenetic analysis. Members of *Botryosphaeriales* have worldwide distribution on many different host plants [3,4,5,6,7,8] and occur as endophytes, pathogens, and saprobes. As opportunistic pathogens, they are of considerable importance to agriculture, horticulture, and forestry [9,10] while causing severe diseases of economically important crops and plants, leading to huge economic losses [11,12].

The family *Botryosphaeriaceae* was established by Theissen and Sydow [13] to accommodate three genera, *Botryosphaeria*, *Dibotryon*, and *Phaeobotryon*, with *Botryosphaeria* as the type genus [1]. Subsequently, Kirk et al. [14] estimated 26 genera within the family *Botryosphaeriaceae*, while Liu et al. [4] reevaluated the family and recognized 29 genera. Up to date, 22 genera with more than 200 species are accepted within the family, based on morphology and molecular evidence [2,15,16,17,18].

During an investigation of *Botryosphaeriaceae* diversity in China and Thailand, a collection of 16 *Botryosphaeriaceae*-like isolates was obtained from several arial parts of the decaying woody hosts. A multi-gene phylogeny based on combined ITS, LSU, *tef1-α*, and *tub2*, coupled with morphological comparisons, was carried out to confirm the taxonomic placement. Additionally, this study extended the taxonomic framework of *Botryosphaeriaceae* by discovering new species, new host records, and new geographical records in China and Thailand.

## 2. Materials and Methods

### 2.1. Specimen Collection, Examination, and Single Spore Isolation

Samples of decaying woody plants were collected from Chiang Rai and Chiang Mai Provinces in Thailand and from Sichuan Province in China between June 2019 and November 2020. Samples were brought to the laboratory by placing them in brown craft paper bags, and the sampling information (date, host, place, GPS, etc.) was recorded.

Morphological observations of fungal structures were made using a LEICA EZ4 dissecting microscope following the method described in Chomnunti et al. [19]. The fungal structures were transferred to a small drop of double distilled water on a clean slide and covered with a glass coverslip. Photomicrographs of the fungal specimens were captured using a Nikon ECLIPSE Ni compound microscope fitted with a Nikon DS-Ri2 digital camera. Macro-morphological structures were photographed with a Discovery V.8 stereo microscope fitted with a CARL ZEISS Axio Cam ERc5S microscope camera. All measurements were made with the Tarosoft (R) Image Frame Work (IFW) program [20], and the images were processed with Adobe Photoshop CC extended version 21.1.2.

Single spore isolations were carried out as described by Chomnunti et al. [19], and fruiting body contents were transferred to a drop of sterile water on a sterilized spot plate. This spore suspension was spread over the Petri dishes containing potato dextrose agar (PDA) and incubated at 25 °C for 12 to 24 h. Germinated spores were transferred onto fresh PDA media plates. These culture plates were incubated at 25 °C in incubators, and colony characteristics were observed and recorded after one week following the method described in Rayner [21]. A total of 56 isolates have been obtained, among which 16 isolates belong to *Botryosphaeriaceae*. In this study, we focus only on the fungal taxa of *Botryosphaeriaceae*. To induce sporulation, cultures were transferred onto fresh PDA media plates using sterile toothpicks or pine needles. The induction results were observed after incubating under near-ultraviolet light for 14–30 d at 25 °C.

Herbarium specimens were deposited in the herbarium of Mae Fah Luang University (MFLU), Chiang Rai, Thailand, and Guizhou Academic of Agriculture Sciences (GZAAS), China. Axenic cultures were deposited in Mae Fah Luang University Culture Collection (MFLUCC) and Guizhou Culture Collection (GZCC). In deciding whether we have new species, we followed the papers of Chethana et al. [22] and Pem et al. [23]. The descriptions are added to the GMS database [24].

### 2.2. DNA Extraction, PCR Amplification and Sequencing

In a sterile environment, fungal mycelium (about 50–100 mg) was scraped using a sterilized toothpick from the colonies grown on PDA media at 25 °C for 2 weeks and then transferred to sterilized 1.5 mL microcentrifuge tubes and maintained at −20 °C for long term storage. Ezup Column Fungi Genomic DNA Purification Kit (Sangon Biotech, Shanghai, China) was used to extract DNA according to the manufacturer’s instructions. The amplifications were performed in a 25 μL reaction volume containing 8.5 μL ddH_2_O, 12.5 μL 2 × PCR Master Mix (Green) (TsingKe Co., Beijing, China), 2 μL of DNA template, and 1 μL of each primer. The genes, primers, and amplification conditions used in this study are listed in Table 1. The PCR products were analyzed by 1.2% agarose gels containing the Safeview DNA stain and sent to Tsingke Biotechnology Co., Ltd. (Chengdu, China) for sequencing.

### 2.3. Sequence Alignment and Phylogenetic Analysis

**Figure 1:**
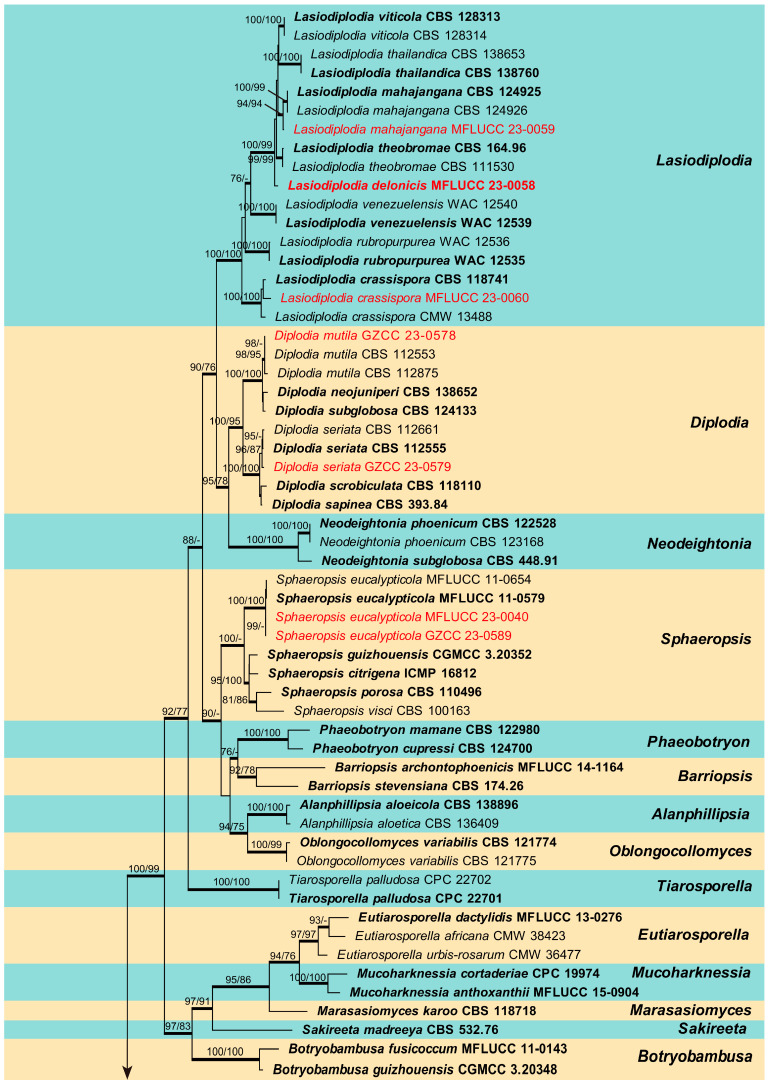

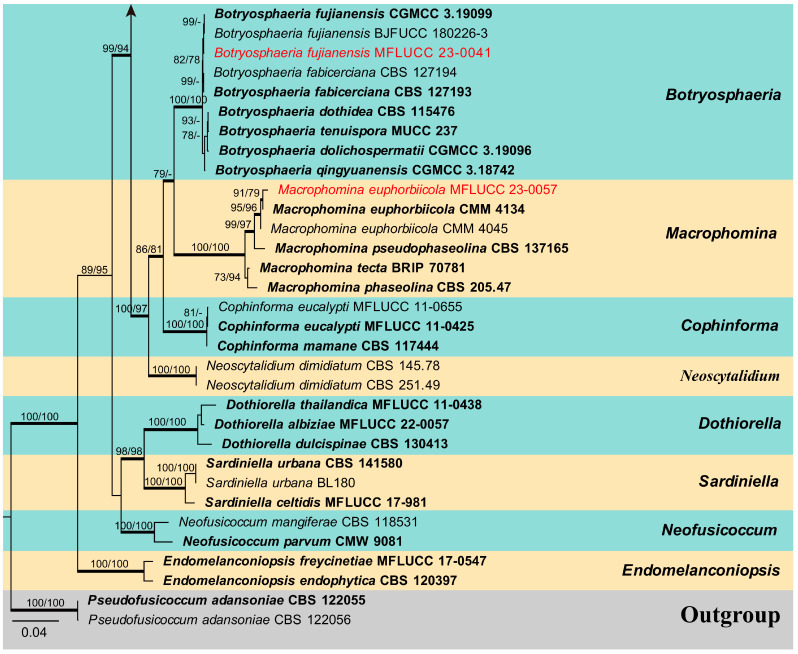
Phylogenetic tree generated from maximum likelihood (ML) analysis based on combined ITS, LSU, *tef1-α*, and *tub2* sequence data for selected closely related genera within the family *Botryosphaeriaceae*. Bootstrap values for maximum likelihood (ML) and maximum parsimony (MP) equal to or greater than 75% are given near the nodes. Bayesian posterior probabilities (BYPP) equal to or greater than 0.95 are denoted in thickened branches. The strain numbers are given after the species names, and ex-type strains are indicated in bold. The newly generated isolates of this study are in red. The tree is rooted with two isolates of *Pseudofusicoccum adansoniae* (CBS 122055, CBS 122056).

**Figure 2:**
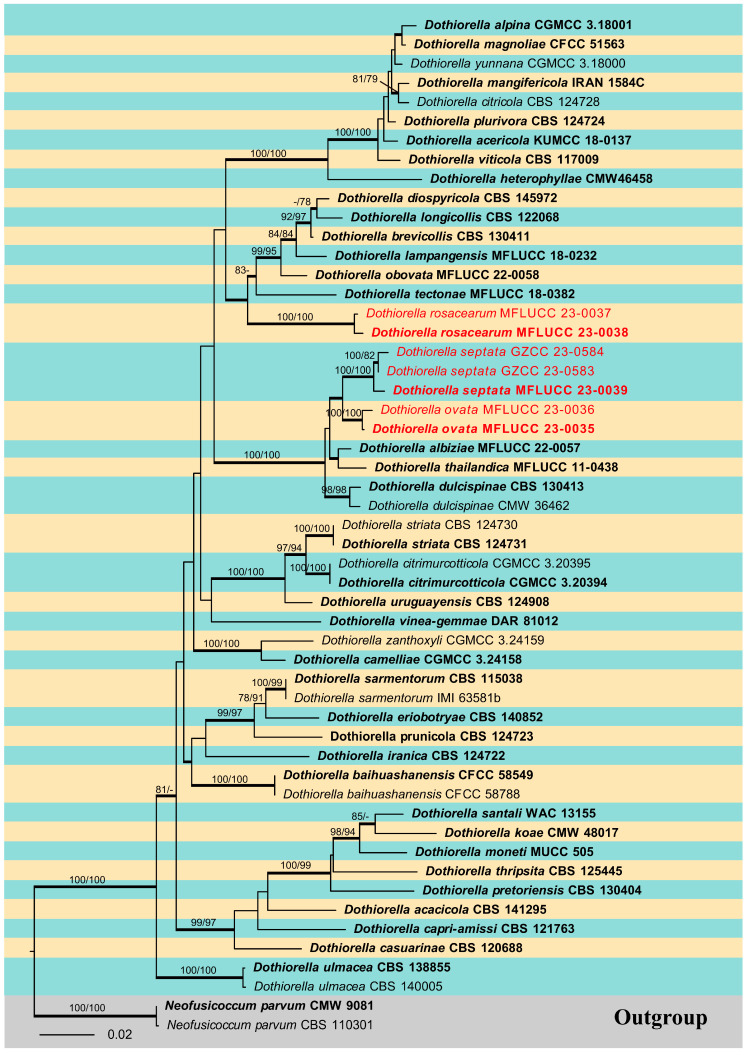
Phylogenetic tree generated from the maximum likelihood (ML) analysis based on combined ITS, LSU, *tef1-α*, and *tub2* sequence data of *Dothiorella*. Bootstrap values for maximum likelihood (ML) and maximum parsimony (MP) equal to or greater than 75% are given near the nodes. Branches with Bayesian posterior probabilities (BYPP) equal to or greater than 0.95 are thickened. The new isolates obtained in this study are indicated in red, and ex-type strains are in bold. The tree is rooted to *Neofusicoccum parvum* (CBS 110301, CMW 9081).

Sequences generated in this study were checked and assembled using BioEdit v.7.0.9 [29] to assure the sequence quality. The closest taxa to the strains obtained in this study were determined with standard nucleotide BLAST searches in NCBI (http://www.ncbi.nlm.nih.gov/, accessed on 20 July 2022). According to the BLAST results and previous literature, appropriate sequences were determined and downloaded from GenBank to construct phylogenetic analysis. Two phylogenetic trees were constructed, one for the whole family *Botryosphaeriaceae* (Figure 1) and the other for the genus *Dothiorella* (Figure 2). Details of the isolates used in this study are listed in Table 2, where two strains of *Pseudofusicoccum adansoniae* (CBS 122055, CBS 122056) and *Neofusicoccum parvum* (CBS 110301, CMW 9081) were selected as the outgroup taxa for *Botryosphaeriaceae* analyses and *Dothiorella* analysis respectively. The sequences were aligned using MAFFT v.7 online (https://mafft.cbrc.jp/alignment/server/, accessed on 1 August 2023) and AliView [30], and the results were checked using BioEdit [29] and manually edited where necessary. The concatenation of different genes was conducted using SequenceMatrix 1.8 [31]. The NEXUS and Phylip files for phylogenetic analyses were obtained using AliView [30]. Phylogenetic analyses of the combined sequence data were performed using maximum likelihood (ML), maximum parsimony (MP), and Bayesian inference (BI) methods, as detailed in Dissanayake et al. [32]. The best model of evolution was determined using MrModeltest v2 [33]. The ML analysis was accomplished using RAxML GUI v. 1.3.1 [34], the MP analysis was performed using PAUP v.4.0b10 [35], and the BI analysis was conducted in MrBayes v 3.2.6 [36]. Phylogenetic trees were visualized with FigTree v.1.4.0 (http://tree.bio.ed.ac.uk/software/figtree/, accessed on 11 August 2023) and further edited in Adobe Illustrator 2020 (Adobe Systems Inc., Lehi, UT, USA). The final alignment was submitted to Figshare (https://figshare.com, at https://doi.org/10.6084/m9.figshare.24187500, accessed on 22 September 2023).

## 3. Results

### 3.1. Phylogenetic Analysis

The combined ITS, LSU, *tef1-α*, and *tub2* sequence dataset of *Botryosphaeriaceae* analysis comprises 89 taxa, including two outgroup taxa. The aligned dataset comprised 2241 characters (ITS: 1–540; LSU: 541–1394; *tef1-α*: 1395–1793; *tub2*: 1794–2241) including gaps. The maximum parsimonious dataset consisted of 2241 variable characters, of which 1470 were constant, 655 were parsimony-informative, and 115 were parsimony-uninformative. The MP analysis resulted in a tree length of 2775 steps [consistency index (CI) = 0.444, retention index (RI) = 0.843, relative consistency index (RC) = 0.374, homoplasy index (HI) = 0.556]. The RAxML analysis of the combined data set yielded a best-scoring tree (Figure 1) with a final ML optimization likelihood value of −17,381.405163. The matrix had 1017 distinct alignment patterns, with 25.04% of undetermined characters or gaps. Estimated base frequencies were: A = 0.218861, C = 0.278646, G = 0.277782, T = 0.224710; substitution rates AC = 1.166395, AG = 2.628375, AT = 1.104924, CG = 1.478465, CT = 4.949967, GT = 1.000000; gamma distribution shape parameter (alpha) = 0.207919. The maximum likelihood (ML), maximum parsimony (MP), and Bayesian methods (BI) for phylogenetic analyses resulted in trees with similar topologies. According to the results of phylogenetic analysis, 16 isolates obtained in this study were grouped into 11 clades and located in the genera *Botryosphaeria*, *Diplodia*, *Dothiorella*, *Lasiodiplodia*, *Macrophomina* and *Sphaeropsis* (Figure 1).

The phylogenetic analysis for the genus *Dothiorella* was carried out, and a phylogenetic tree combining ITS, LSU, *tef1-α*, and *tub2* sequence data was also constructed (Figure 2). This dataset included 53 ingroup taxa and two outgroup taxa. The aligned dataset comprised 2062 characters (ITS: 1–514; LSU: 515–1331; *tef1-α*: 1332–1630; *tub2*: 1631–2062) including gaps. The maximum parsimonious dataset consisted of 2066 variable characters, of which 1637 were constant, 328 were parsimony-informative, and 101 were parsimony-uninformative. The MP analysis resulted in a tree length of 1223 steps [consistency index (CI) = 0.517, retention index (RI) = 0.760, relative consistency index (RC) = 0.393, homoplasy index (HI) = 0.483]. In the ML analyses, the best-scoring RAxML tree with a final likelihood value of −9705.573604 is presented. The matrix had 606 distinct alignment patterns, with 25.62% of undetermined characters or gaps. Estimated base frequencies were: A = 0.215885, C = 0.284638, G = 0.269261, T = 0.230216; substitution rates AC = 1.152119, AG = 2.141304, AT = 1.169569, CG = 1.149449, CT = 5.009593, GT = 1.000000; gamma distribution shape parameter (alpha) = 0.143638. The maximum likelihood (ML), maximum parsimony (MP), and Bayesian methods (BI) for phylogenetic analyses resulted in trees with similar topologies, and the result of ML analysis is shown in Figure 2. Phylogenetic results showed that the isolates obtained in this study were nested within the genus *Dothiorella* and grouped into three clades as distinct species.

### 3.2. Taxonomy

**Figure 3:**
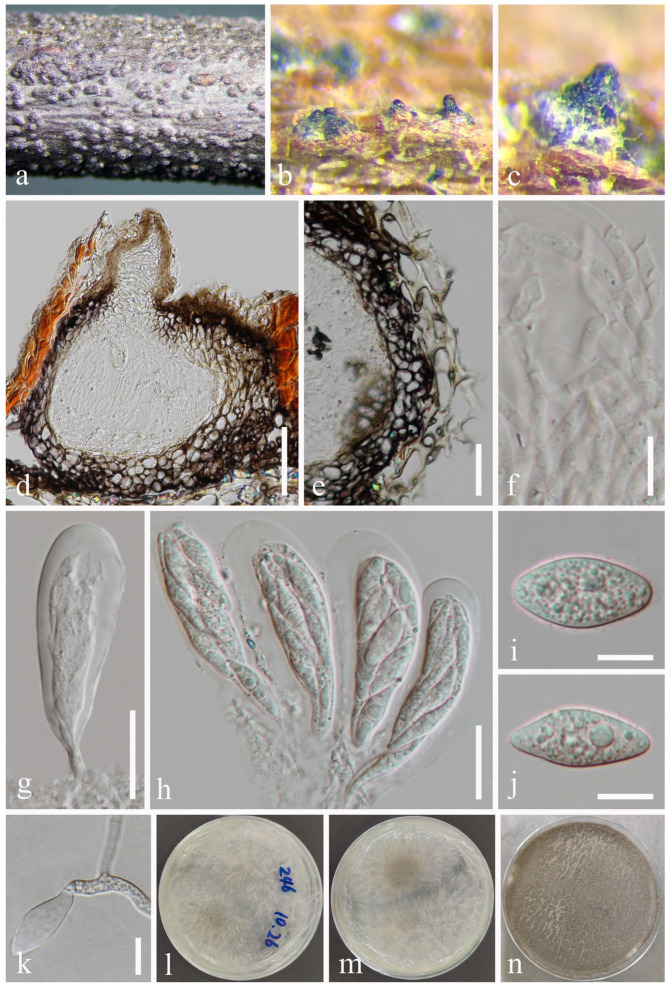
*Botryosphaeria fujianensis* (MFLU 23-0012). (**a**–**c**) Ascomata on host substrate. (**d**) Vertical section of ascomata. (**e**) Structure of the peridium. (**f**) Pseudoparaphyses. (**g**,**h**) Asci. (**i**,**j**) Ascospores. (**k**) Germinated ascospore. (**l**–**n**) Colonies on PDA, above (**l**,**n**) and below (**m**). Scale bars: (**d**) = 50 μm, (**e**) = 5 μm, (**f**–**j**) = 10 μm, (**k**) = 50 μm.

*Botryosphaeria fujianensis* Z.P. Dou, W. He & Y. Zhang, Mycosystema 40: 482 [37] Figure 3.

Index Fungorum number: IF826938.

*Saprobic* on twigs of *Tectona grandis* L.F. Sexual morph: *Ascomata* 140–216 × 178–278 μm ($\bar{x}$ = 182 × 211 μm, *n* = 20), well-developed, bursting through the bark, generally emerged from the host surface, scattered or gregarious, black, subglobose to obpyriform. *Peridium* 16–56 μm wide, composed of five strata; an outer stratum has black, thick-walled cells, and the middle and inner layers have black, thin-walled cells. *Hamathecium* comprising up to 3–5 μm wide, dense, cellular pseudoparaphyses, anastomosing above and between the asci. *Asci* 76–125 × 20–33 μm (*n* = 20), bitunicate, eight-spored, broadly clavate, apically rounded and with a visible ocular chamber. *Ascospores* 20–27 × 7–11 μm ($\bar{x}$ = 24 × 10 μm, *n* = 50), initially rhomboid, hyaline, aseptate, elliptical to ovoid, thick-walled, smooth at maturity, apiculus at either end. Asexual morph: Not observed.

Culture characteristics: Ascospores germinating on PDA within 12 h. Colonies are fast growing on PDA, reaching 60 mm diam. after 7 d at 20–25 °C. Circular, white in first few days, becoming pale grey from the center after two weeks, and finally black after three weeks, felt-like, flattened, surface smooth.

Material examined: Thailand, Chiang Mai Province, Amphoe Mae Taeng, Tambon Pa Pae, 19°06′32.6″ N, 98°44′21.1″ E, on dead twigs of *Tectona grandis* (*Lamiaceae*), 8 August 2019, Na Wu, YW246 (MFLU 23-0012), living culture MFLUCC 23-0041.

Notes: The phylogenetic results (Figure 1) showed that our newly obtained isolate clustered together with *Botryosphaeria fujianensis*, and we identified it as *B*. *fujianensis*. There was only the asexual morph provided when Chu et al. [37] described this species, and we illustrate the sexual morph of *B. fujianensis* and reported it as the new record from Thailand in this study.

**Figure 4:**
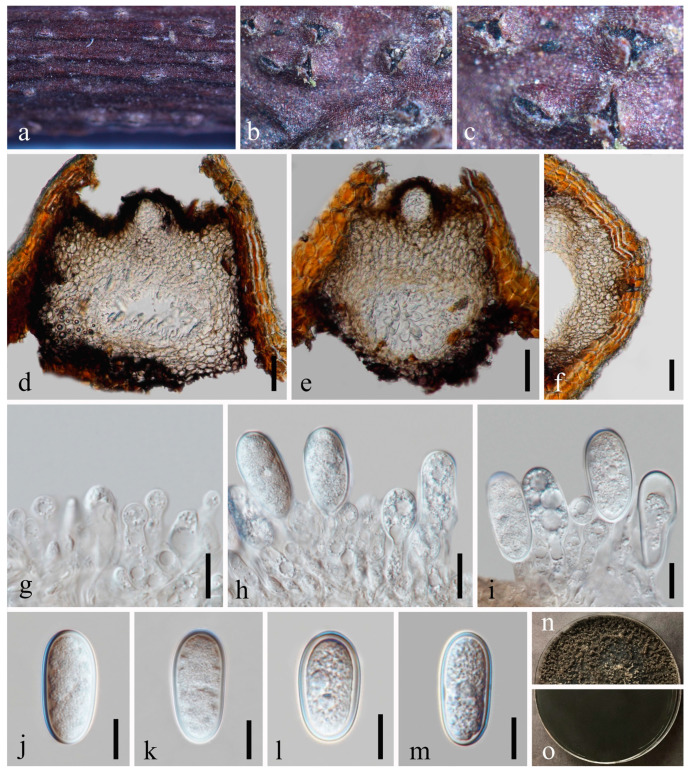
*Diplodia mutila* (GZAAS 23-0582). (**a**–**c**) Conidiomata on host substrate. (**d**,**e**) Vertical section of conidiomata. (**f**) Section of peridium. (**g**–**i**) Conidiogenous cells and developing conidia. (**j**–**m**) Conidia. (**n**,**o**) Colonies on PDA, above (**n**) and below (**o**). Scale bars: (**d**–**f**) = 20 μm, (**g**–**m**) = 10 μm.

*Diplodia mutila* Fries in Montagne, Ann. Sci. nat., sér. 21: 302 [38] Figure 4.

Index Fungorum number: IF201741; Facesoffungi number: FoF00147.

*Saprobic* on dead twigs of *Prunus persica* L. Sexual morph: Not observed. Asexual morph: *Conidiomata* 213–306 × 247–313 μm ($\bar{x}$ = 266 × 282 μm, *n* = 20), semi-immersed or immersed in the substrate, dark brown to black, solitary, globose to ovoid, centrally ostiolate. *Ostiole* 28–45 μm diam., centrally located, short papillate. *Peridium* up to 53–78 μm wide, 5–7 layers, consisting of brown and small-celled *textura angularis*. *Conidiophores* are reduced to conidiogenous cells. *Conidiogenous cells* 8–14 × 3–7 μm ($\bar{x}$ = 9 × 4 μm, *n* = 20), hyaline, cylindrical, discrete, smooth-walled, slightly swollen at the base, forming a single conidium at the tip. *Conidia* 23–29 × 12–15 μm ($\bar{x}$ = 27 × 14 μm, *n* = 50), hyaline, aseptate, externally smooth, internally verruculose, thick-walled, oblong to ovoid, straight, both ends broadly rounded.

Culture characteristics: Conidia germinating on PDA within 24 h. Colonies are fast growing at 20–25 °C, becoming ash-grey on the surface after 7 days and finally black after two weeks, felt-like, dense, convex with the papillate surface, aerial.

Material examined: China, Sichuan Province, Chengdu City, High Tech West Zone, Xi Yuan Avenue, University of Electronic Science and Technology of China campus, 30°45’9″ N, 103°55’31″ E, on dead twigs of *Prunus persica* L., 22 November 2020, H.Z. Du, YW290 (GZAAS 23-0582), living culture GZCC 23-0578.

Notes: We identified our isolate as *Diplodia mutila* based on morphology and phylogeny. This is the first record of *Di. mutila* found on *Prunus persica* in China.

**Figure 5:**
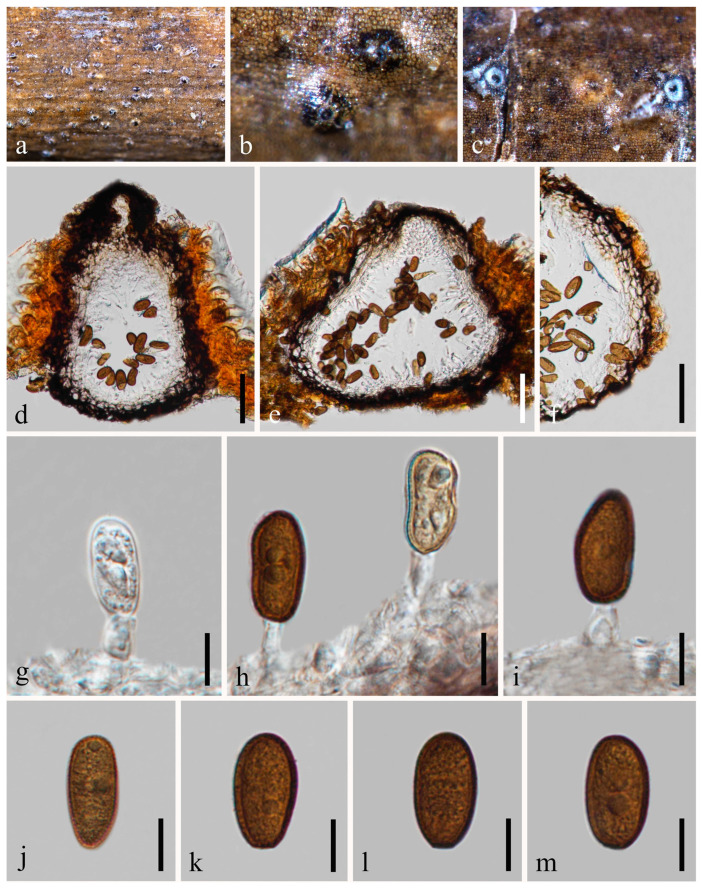
*Diplodia seriata* (GZAAS 23-0583). (**a**–**c**) Conidiomata on host substrate. (**d**,**e**) Vertical section of conidiomata. (**f**) Section of peridium. (**g**–**i**) Conidiogenous cells and developing conidia. (**j**–**m**) Conidia. Scale bars: (**d**–**f**) = 20 μm, (**g**–**m**) = 10 μm.

*Diplodia seriata* De Not., Mém. R. Accad. Sci. Torino, Ser. 27: 26 [39] Figure 5.

Index Fungorum number: IF180468.

*Saprobic* on dead twigs of *Wisteria sinensis* L. Sexual morph: Not observed. Asexual morph: *Conidiomata* 138–245 × 206–240 μm ($\bar{x}$ = 175 × 228 μm, *n* = 20), semi-immersed or immersed in the substrate, dark brown to black, solitary, pyriform, initially intraepidermal, visible as black dots on the host when mature, thick-walled, glabrous, centrally ostiolate. *Ostiole* 16–30 μm diam., centrally located, short papillate. *Peridium* up to 12–30 μm wide, 3–5 layers, consisting of brown and small-celled *textura angularis*, becoming hyaline towards the inner region. *Conidiophores* are reduced to conidiogenous cells. *Conidiogenous cells* 6–13 × 3–5 μm ($\bar{x}$ = 8 × 4 μm, *n* = 20), hyaline, cylindrical, discrete, smooth-walled, slightly swollen at the base, forming a single conidium at the tip. *Conidia* 18–27 × 9–13 μm ($\bar{x}$ = 22 × 11 μm, *n* = 50), brown, aseptate, externally smooth, internally verruculose, subcylindrical to ellipsoid, moderately thick-walled, ends rounded, often with a truncate base.

Culture characteristics: Conidia germinating on PDA within 24 h. Colonies are fast growing at 20–25 °C, becoming ash-grey on the surface after 7 days, and finally black after two weeks, velvety and floccose, dense, convex with papillate surface, aerial.

Material examined: China, Sichuan Province, Chengdu City, High Tech West Zone, Xi Yuan Avenue, University of Electronic Science and Technology of China campus, 30°45’9″ N, 103°55’31″ E, on dead twigs of *Wisteria sinensis* L., 22 November 2020, H.Z. Du, YW297 (GZAAS 23-0583), living culture GZCC 23-0579.

Notes: The sexual morph of *Diplodia seriata* is linked to *Botryosphaeria obtusa* Shoemaker [40]. We identified our isolate as *Di. seriata* based on morphology and phylogeny. This is the first record of *Di. seriata* found on *Wisteria sinensis* in China.

**Figure 6:**
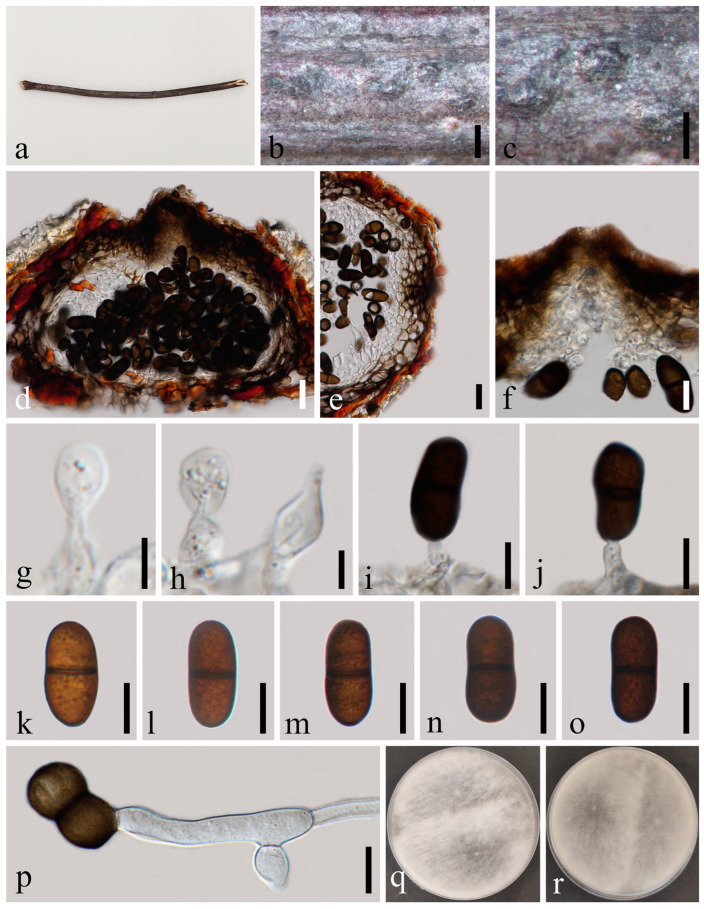
*Dothiorella ovata* (MFLU 23-0009, holotype). (**a**–**c**) Conidiomata on host substrate. (**d**) Vertical section of conidiomata. (**e**) Section of peridium. (**f**) Ostiolar region with periphyses. (**g**–**j**) Conidiogenous cells and developing conidia. (**k**–**o**) Conidia. (**p**) Germinated conidium. (**q**,**r**) Colonies on PDA, above (**q**) and below (**r**). Scale bars: (**b**,**c**) = 50 μm, (**d**,**e**) = 20 μm, (**f**) = 10 μm, (**g**,**h**) = 5 μm, (**i**–**p**) = 10 μm.

*Dothiorella ovata* N. Wu, A.J. Dissanayake & Jian K. Liu sp. nov. Figure 6.

Index Fungorum number: IF900578; Facesoffungi number: FoF14258.

Etymology: In reference to the ovoid conidia.

Holotype: MFLU 23-0009.

*Saprobic* on a dead wood. Sexual morph: Not observed. Asexual morph: *Conidiomata* 119–173 × 160–273 μm ($\bar{x}$ = 141 × 204 μm, *n* = 20), semi-immersed or immersed in the substrate, emerging through the epidermis when mature, globose to subglobose, pyriform, dark brown, unilocular, solitary, glabrous, ostiolate. *Ostiole* 22–26 μm diam., single, circular, papillate, centrally located. *Peridium* up to 16–38 μm wide, comprising host and fungal tissues, thick-walled, dark brown to hyaline cells of *textura angularis*. Paraphyses absent. *Conidiophores* are reduced to conidiogenous cells. *Conidiogenous cells* 4–11 × 3–4 μm ($\bar{x}$ = 7 × 4 μm, *n* = 20), hyaline, phialidic, cylindrical, straight or curved, smooth-walled. *Conidia* 20–26 × 9–11 μm ($\bar{x}$ = 22 × 10 μm, *n* = 50), hyaline, aseptate, ovoid, rounded at both ends or sometimes with truncate bases, becoming pigmented brown and one septate at maturation, constricted in the middle, smooth-walled, without longitudinal striations or mucilaginous sheath.

Culture characteristics: Conidia germinating on PDA within 12 h. Colonies are fast growing on PDA, reaching 90 mm diam. after 5–6 days at 20–23 °C. Sparse, aerial, filamentous, white in first few days, after 2 weeks, becoming black.

Material examined: Thailand, Chiang Mai Province, Amphoe Mae Taeng, Tambon Cho Lae, 19°08’01.3″ N, 99°00’29.4″ E, on an unidentified dead wood, 6 August 2019, Na Wu, YW177 (MFLU 23-0009, holotype); ex-type living culture MFLUCC 23-0035; *ibid*., 7 August 2019, Na Wu, YW231 (MFLU 23-0010, paratype), living culture MFLUCC 23-0036.

Notes: *Dothiorella ovata* is nested in between *Do. albiziae*, *Do. septata* and *Do. thailandica* but can be recognized as a distinct lineage (Figure 2). Morphologically, *Do. ovata* is similar to *Do. septata* and *Do. albiziae*, which in having oblong to ovoid, hyaline conidia becoming brown and one septate at maturation. However, *Do. ovata* differs from *Do. septata* and *Do. albiziae* by its slightly constricted septum and the larger conidia. In addition, a comparison of *tef1-α* sequences data of *Do. ovata* and *Do. albiziae* showed that there are 14 bp (base pair) differences (of 252 bp including the gaps), while *Do. ovata* and *Do. thailandica* showed 18 bp differences (of 302 bp including the gaps). Therefore, we introduce *Do. ovata* as a new species.

**Figure 7:**
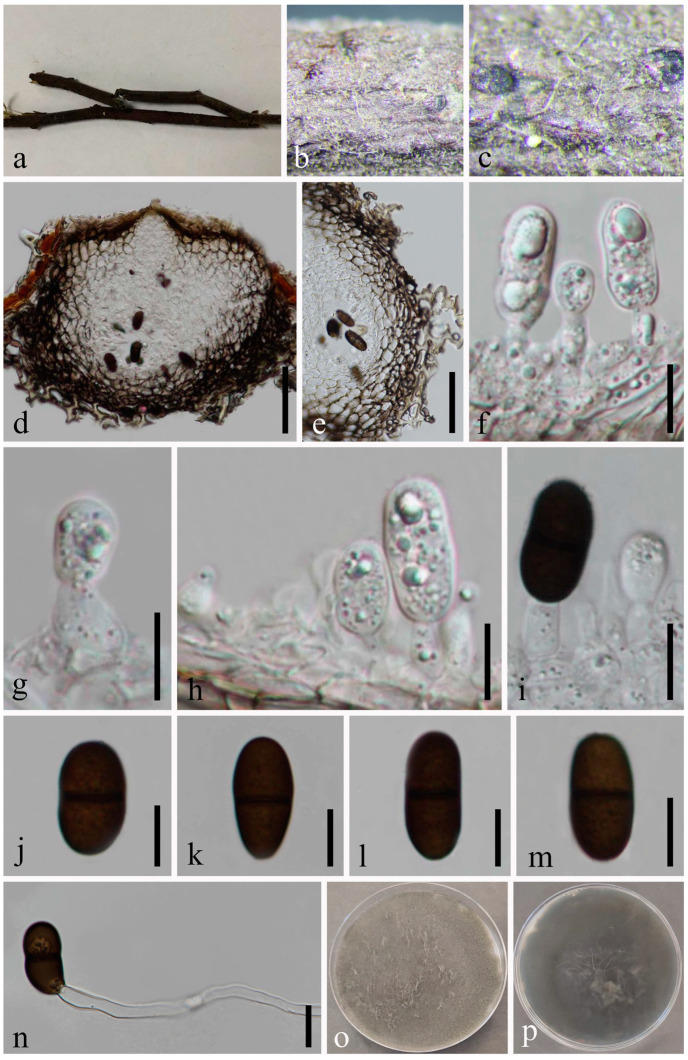
*Dothiorella rosacearum* (MFLU 23-0014, holotype). (**a**–**c**) Conidiomata on host substrate. (**d**) Vertical section of conidiomata. (**e**) Section of peridium. (**f**–**i**) Conidiogenous cells and developing conidia. (**j**–**m**) Conidia. (**n**) Germinated conidium. (**o**,**p**) Colonies on PDA, above (**o**) and below (**p**). Scale bars: (**d**,**e**) = 50 μm, (**f**–**n**) = 10 μm.

*Dothiorella rosacearum* N. Wu, A.J. Dissanayake & Jian K. Liu, sp. nov. Figure 7.

Index Fungorum number: IF900579; Facesoffungi number: FoF14259.

Etymology: Referring to the host family Rosaceae on which the type specimen was collected.

Holotype: MFLU 23-0014.

*Saprobic* on dead twigs of *Amygdalus* sp. (*Rosaceae*). Sexual morph: Not observed. Asexual morph: *Conidiomata* 153–234 × 222–336 μm ($\bar{x}$ = 187 × 270 μm, *n* = 20), immersed, scattered, dark brown to black, globose or subglobose, solitary, initially intraepidermal, visible as black dots on the host when mature, thick-walled, glabrous. *Ostiole* 30–32 μm diam., single, straight, sometimes bent, centrally located. *Peridium* up to 31–45 μm wide, comprising host and fungal tissues, dark brown to black, 5–10 cell layers of *textura angularis*, becoming thin-walled and hyaline towards the inner region. *Paraphyses* absent. *Conidiophores* are reduced to conidiogenous cells. *Conidiogenous cells* 4–9 × 3–5 μm ($\bar{x}$ = 7 × 4 μm, *n* = 20), hyaline, aseptate, contents granular, cylindrical to ellipsoidal, smooth-walled, unbranched or occasionally branched, with swollen bases. *Conidia* 17–21 × 7–10 μm ($\bar{x}$ = 18 × 8 μm, *n* = 50), hyaline, aseptate, contents granular, smooth, thin-walled, oval, straight, rounded at the apex, or sometimes with truncate bases, becoming brown and septate when aged, without longitudinal striations or mucilaginous sheath.

Culture characteristics: Conidia germinating on PDA within 12 h. Colonies are fast growing on PDA, reaching 90 mm diam. after 5–6 days at 20–23 °C. Sparse, aerial, filamentous, becoming dark brown to black after 2 weeks.

Material examined: Thailand, Chiang Mai Province, Amphoe Mae Taeng, Tambon Pa Pae, 19°06’32.6″ N, 98°44’21.1″ E, on dead twigs of *Amygdalus* sp. (*Rosaceae*), 8 August 2019, Na Wu, YW255 (MFLU 23-0014, holotype); ex-type living culture MFLUCC 23-0038; *ibid.*, on an unidentified decaying wood, 7 August 2019, Na Wu, YW253 (MFLU 23-0013, paratype), living culture MFLUCC 23-0037.

Notes: The phylogenetic result (Figure 2) showed that two isolates of *Dothiorella rosacearum* constitute a distinct lineage but claded closer to *Do. brevicollis*, *Do. diospyricola*, *Do. lampangensis*, *Do. longicollis*, *Do. obovata* and *Do. tectonae*. A comparison of ITS sequences data between *Do. rosacearum* and *Do. tectonae* showed that there are 19 bp of 539 base pairs differences (including the gaps). In addition, the shortly raised irregular striations can be found on the conidia of *Do. tectonae*, while no striations were observed in *Do. rosacearum*. Therefore, *Do*. *rosacearum* is a morphologically and phylogenetically distinct species and herein introduced as a new species.

**Figure 8:**
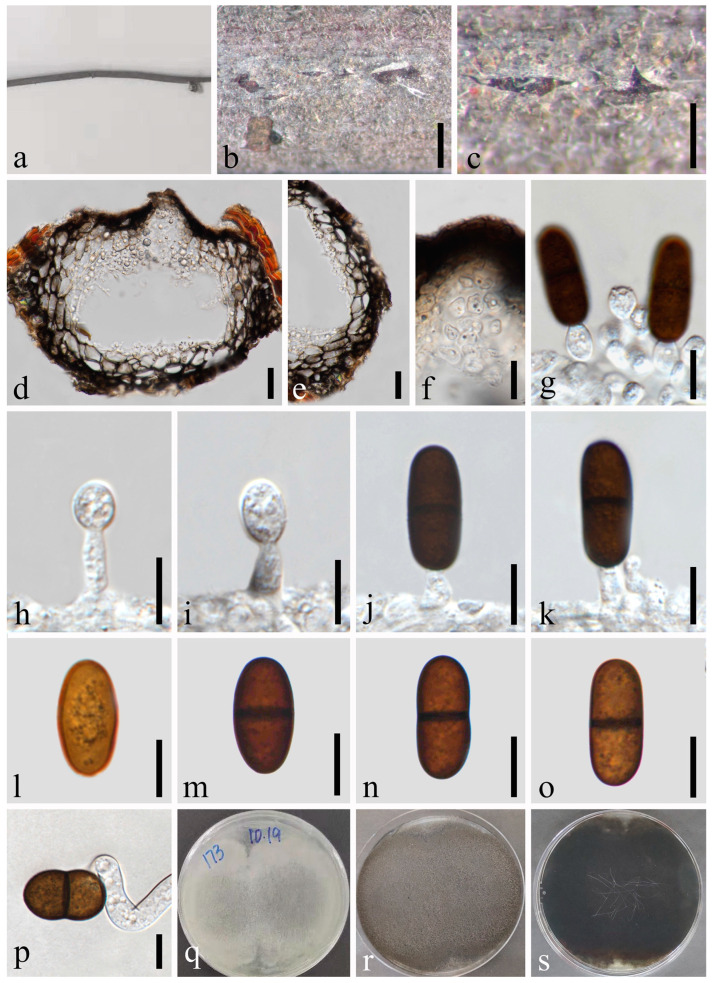
*Dothiorella septata* (MFLU 23-0007, holotype). (**a**–**c**) Conidiomata on host substrate. (**d**) Vertical section of conidiomata. (**e**) Section of peridium. (**f**) Ostiolar region with periphyses. (**g**–**k**) Conidiogenous cells and developing conidia. (**l**–**o**) Conidia. (**p**) Germinated conidium. (**q**–**s**) Colonies on PDA, above (**q**,**r**) and below (**s**). Scale bars: (**b**) = 50 μm, (**c**,**d**) = 20 μm, (**e**–**p**) = 10 μm.

*Dothiorella septata* N. Wu, A.J. Dissanayake & Jian K. Liu, sp. nov. Figure 8.

Index Fungorum number: IF900580; Facesoffungi number: FoF14260.

Etymology: The epithet “septata” refers to the septum observed in mature conidia.

Holotype: MFLU 23-0007.

*Saprobic* on an unidentified dead wood. Sexual morph: Not observed. Asexual morph: *Conidiomata* 114–139 × 150–198 μm ($\bar{x}$ = 126 × 172 μm, *n* = 20), pyriform or subglobose, immersing through the host epidermis, unilocular, glabrous, thick-walled, ostiolate. *Ostiole* 19–25 μm diam., single, straight, centrally located. *Peridium* up to 16–303 μm wide, with outer 3–5 layers of brown cells of *textura angularis* and inner 1–2 layers of hyaline cells of *textura angularis*. *Paraphyses* absent. *Conidiophores* are reduced to conidiogenous cells. *Conidiogenous cells* 5–10 × 2–5 μm ($\bar{x}$ = 7 × 3 μm, *n* = 20), hyaline, phialidic, subcylindrical, smooth-walled. *Conidia* 19–21 × 8–10 μm ($\bar{x}$ = 21 × 9 μm, *n* = 50), oblong to ovoid with a broadly rounded apex, initially hyaline to yellowish and aseptate, becoming brown to dark brown and one septate at maturation, slightly constricted at the septum, smooth-walled, without a mucilaginous sheath.

Culture characteristics: Conidia germinating on PDA within 12 h. Colonies are fast growing on PDA, reaching 90 mm diam. after 5–6 days at 20–23 °C. Sparse, aerial, filamentous, smooth with a crenate edge, white in first few days, becoming grey after one week, and after 2 weeks, becoming black.

Material examined: Thailand, Chiang Mai Province, Amphoe Mae Taeng, Tambon Sop Poeng, 19°07’52.3″ N, 98°45’35.7″ E, on an unidentified dead wood, 9 August 2019, Na Wu, YW173 (MFLU 23-0007, holotype); ex-type living culture MFLUCC 23-0039; *ibid*., on a decaying wood in a mountain, 7 August 2019, Na Wu, YW217 (GZAAS 23-0587, paratype), living culture GZCC 23-0583; *ibid.*, YW228 (GZAAS 23-0588, paratype), living culture GZCC 23-0584.

Notes: The phylogenetic results (Figure 2) showed that our isolates clustered with *Do. ovata* and formed a sister group. A comparison of ITS and *tef1-α* nucleotides shows that *Do. septata* is significantly different from its sister species, *Do. ovata* by 7/569 bp (1.2%) in ITS and 13/303 bp (4.3%) in *tef1-α*. In the phylogenetic analysis, these two species formed two distinct clades in *Dothiorella*. Morphologically, there are several differences in conidial morphology between these two species. Considering the morpho-molecular data, we introduced *Do*. *septata* as a new species.

**Figure 9:**
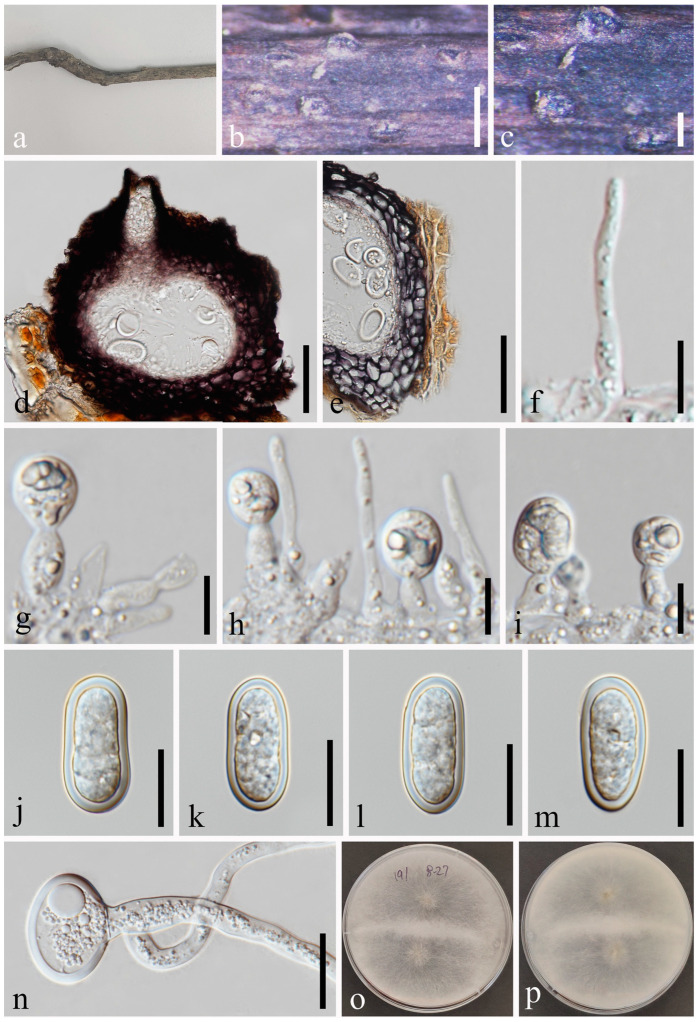
*Lasiodiplodia crassispora* (MFLU 23-0011). (**a**–**c**) Conidiomata on host substrate. (**d**) Vertical section of conidiomata. (**e**) Section of peridium. (**f**) Paraphyses. (**g**–**i**) Conidiogenous cells and developing conidia. (**j**–**m**) Conidia. (**n**) Germinated conidium. (**o**,**p**) Colonies on PDA, above (**o**) and below (**p**). Scale bars: (**b**) = 500 μm, (**c**) = 200 μm, (**d**,**e**) = 50 μm, (**f**–**i**) = 10 μm, (**j**–**n**) = 20 μm.

*Lasiodiplodia crassispora* T.I. Burgess & P.A.Barber, Mycologia 98: 425 [41] Figure 9.

Index Fungorum number: IF500235.

*Saprobic* on an unidentified dead wood. Sexual morph: Not observed. Asexual morph: *Conidiomata* 151–191 × 178–202 μm ($\bar{x}$ = 171 × 188 μm, *n* = 20), semi-immersed or immersed in the substrate, solitary, gregarious or confluent, globose to subglobose, centrally ostiolate. *Ostiole* 21–33 μm diam., centrally located, papillate. *Peridium* up to 19–46 μm wide, consisting of black and small-celled *textura angularis*. *Paraphyses* 2–3 μm wide, hyaline, cylindrical, aseptate, not branched, rounded at apex. *Conidiophores* are reduced to conidiogenous cells. *Conidiogenous cells* 7–12 ×4–7 μm ($\bar{x}$ = 9 × 5 μm, *n* = 20), hyaline, cylindrical. *Conidia* 27–33 × 14–17 μm ($\bar{x}$ = 30 × 16 μm, *n* = 50), hyaline, aseptate, ellipsoid to ovoid, thick-walled, without longitudinal striations or mucilaginous sheath.

Culture characteristics: Conidia germinating on PDA within 24 h. Colonies are fast growing on PDA at 20–25 °C, becoming ash-grey on the surface after 7 days, the reverse is pale grey to grey, and finally black after two weeks, felt-like, sparse, aerial, surface smooth with a crenate edge, filamentous.

Material examined: Thailand, Chiang Mai, Amphoe Mae Taeng, Tambon Cho Lae, 19°08’01.3″ N, 99°00’29.4″ E, on an unidentified dead wood, 6 August 2019, Na Wu, YW191 (MFLU 23-0011), living culture MFLUCC 23-0060.

Notes: The morphology of our collection obtained from decaying woody is similar to the original description of *Lasiodiplodia crassispora* [41]. In the multi-gene phylogenetic analysis, our new collection clustered with the ex-type strain of *L*. *crassispora* (CBS 118741) with strong bootstrap support, and we identified it as *L*. *crassispora*.

**Figure 10:**
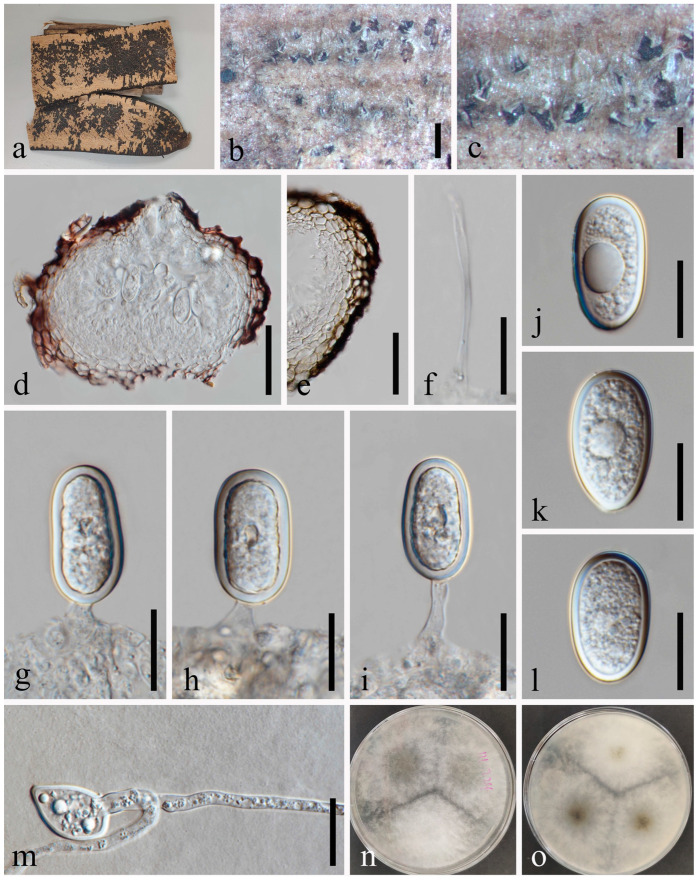
*Lasiodiplodia delonicis* (MFLU 23-0005, holotype). (**a**) Specimen. (**b**,**c**) Conidiomata on host substrate. (**d**) Vertical section of conidiomata. (**e**) Section of peridium. (**f**) Paraphyses. (**g**–**i**) Conidiogenous cells and developing conidia. (**j**–**l**) Conidia. (**m**) Germinated conidium. (**n**,**o**) Colonies on PDA, above (**n**) and below (**o**). Scale bars: (**b**) = 50 μm, (**c**) = 20 μm, (**d**,**e**) = 50 μm, (**f**–**m**) = 20 μm.

*Lasiodiplodia delonicis* N. Wu, A.J. Dissanayake & Jian K. Liu, sp. nov. Figure 10.

Index Fungorum number: IF900581; Facesoffungi number: FoF14261.

Etymology: Referring to the host genus on which the fungus was collected, *Delonix regia* (*Fabaceae*).

Holotype: MFLU 23-0005.

*Saprobic* on a fallen pod of *Delonix regia* L. Sexual morph: Not observed. Asexual morph: *Conidiomata* 110–180 × 124–171 μm ($\bar{x}$ = 139 × 151 μm, *n* = 20), pyriform, immersed to semi-immersed, solitary, black, ostiolate. *Ostiole* 20–31 μm diam., central, cylindrical to subcylindrical. *Peridium* up to 18–35 μm wide, with outer 3–4 layers of brown cells of *textura angularis* and inner 1–2 layers of hyaline cells of *textura angularis*. *Paraphyses* 2–3 μm wide, hyaline, cylindrical, aseptate, not branched. *Conidiophores* are reduced to conidiogenous cells. *Conidiogenous cells* 4–16 × 4–6 μm ($\bar{x}$ = 8 × 5 μm, *n* = 20), hyaline, cylindrical, sometimes slightly curved. *Conidia* 26–38 × 13–29 μm ($\bar{x}$ = 32 × 17 μm, *n* = 50), ellipsoid to ovoid, hyaline, aseptate, thick-walled with granular content, occasionally truncate at base, without longitudinal striations or mucilaginous sheath.

Culture characteristics: Conidia germinating on PDA within 24 h. Colonies are fast growing on PDA, reaching 90 mm diam. after 5 days at 20–25 °C, becoming ash-grey on the surface after one week, with the reverse side of the colonies pale grey to grey, and finally black after two weeks, felt-like, sparse, aerial, surface smooth with crenate edge, filamentous.

Material examined: Thailand, Chiang Rai Province, Amphoe Mueang, Tambon Nang Lae, 20°02’22.7″ N, 99°53’38.1″ E, on a fallen pod of *Delonix regia*, 17 July 2019, Na Wu, YW111 (MFLU 23-0005, holotype); ex-type living culture MFLUCC 23-0058.

Notes: The phylogenetic tree based on ITS, LSU, *tef1-α*, and *tub2* sequence data showed that the new species *Lasiodiplodia delonicis* (Figure 1) is supported by an absolute bootstrap support (ML/MP/BI = 100/100/1.0). Morphologically, *L. delonicis* is distinct from other *Lasiodiplodia* species by its thicker conidial wall and larger conidia. Additionally, conidia of *L. delonicis* are hyaline throughout the life cycle.

**Figure 11:**
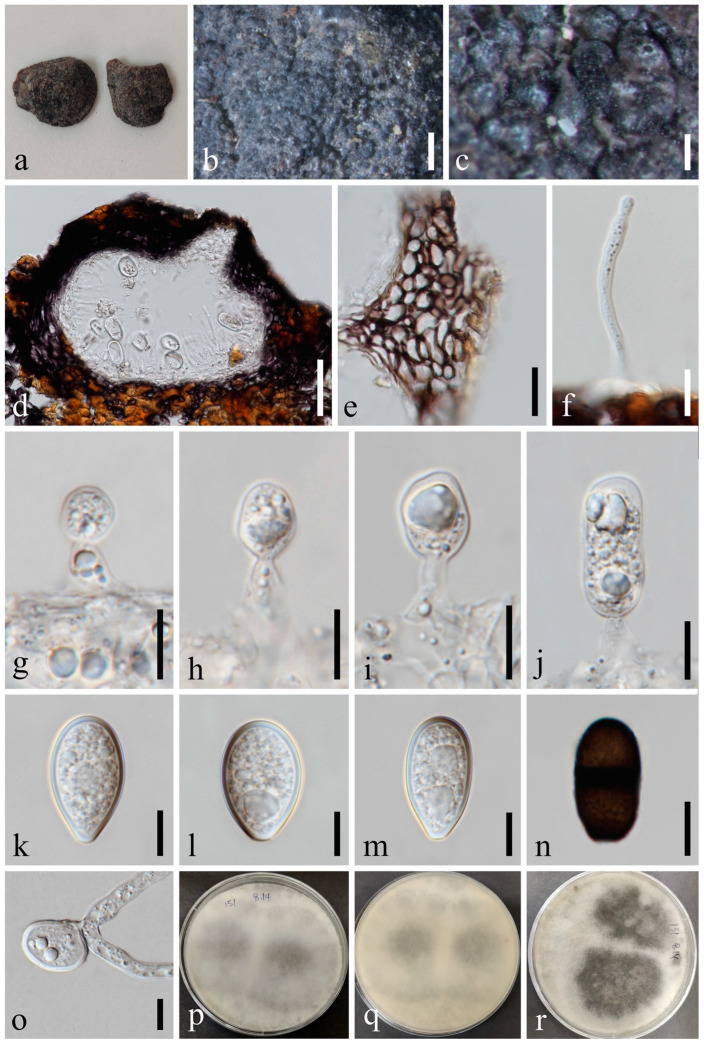
*Lasiodiplodia mahajangana* (MFLU 23-0006). (**a**–**c**) Conidiomata on host substrate. (**d**) Vertical section of conidiomata. (**e**) Section of peridium. (**f**) Paraphyses. (**g**–**j**) Conidiogenous cells and developing conidia. (**k**–**n**) Conidia. (**o**) Germinated conidium. (**p**–**r**) Colonies on PDA, above (**p**,**r**) and below (**q**). Scale bars: (**b**) = 100 μm, (**c**) = 20 μm, (**d**) = 50 μm, (**e**) = 20 μm, (**f**–**o**) = 10 μm.

*Lasiodiplodia mahajangana* Begoude, Jol. Roux & Slippers, Mycol. Progr. 9: 110 [42] Figure 11.

Index Fungorum number: IF514012.

*Saprobic* on dead seeds of *Dipterocarpus retusus* L. Sexual morph: Not observed. Asexual morph: *Conidiomata* 127–164 × 133–192 μm ($\bar{x}$ = 148 × 160 μm, *n* = 20), solitary or compound, superficial or immersed, unilocular or multilocular, globose to subglobose, thick-walled, glabrous, ostiolate. *Ostiole* 26–33 μm diam., single, long, cylindrical to subcylindrical, eccentric. *Peridium* up to 14–23 μm wide, consisting of brown and small-celled *textura angularis*. *Paraphyses* 2–4 μm wide, hyaline, cylindrical, aseptate, not branched, rounded at apex. *Conidiophores* are reduced to conidiogenous cells. *Conidiogenous cells* 6–13 × 4–5 μm ($\bar{x}$ = 8 × 5 μm, *n* = 20), hyaline, cylindrical, proliferating percurrently to form a periclinal thickening. *Conidia* 24–31 × 14–18 μm ($\bar{x}$ = 27 × 16 μm, *n* = 50), initially aseptate, hyaline, ellipsoid to ovoid, thick-walled with granular content, rounded at apex, occasionally truncate at the base, one septate at maturation, without longitudinal striations or mucilaginous sheath.

Culture characteristics: Conidia germinating on PDA within 24 h. Colonies are fast growing on PDA at 20–25 °C, becoming ash-grey on the surface after 7 days, with the reverse side of the colonies pale grey to grey, and finally black after two weeks, felt-like, sparse, aerial, surface smooth with crenate edge, filamentous.

Material examined: Thailand, Chiang Mai Province, Amphoe Mae Taeng, Tambon Cho Lae, 19°08’01.3″ N, 99°00’29.4″ E, on dead seeds of *Dipterocarpus retusus* L., 10 August 2019, Na Wu, YW151 (MFLU 23-0006), living culture MFLUCC 23-0059.

Notes: In the phylogenetic tree, an isolate obtained in this study (MFLUCC 23-0059) grouped with *Lasiodiplodia mahajangana* (Figure 1) (ML/MP/BI = 94/95/1.0). Our sample is morphologically similar to *L*. *mahajangana* as of the report by Begoude et al. [42], having hyaline, aseptate, ellipsoid to ovoid, thick-walled conidia, which becomes one septate after maturation. We identified our collection as *L*. *mahajangana* based on morphology and phylogeny.

**Figure 12:**
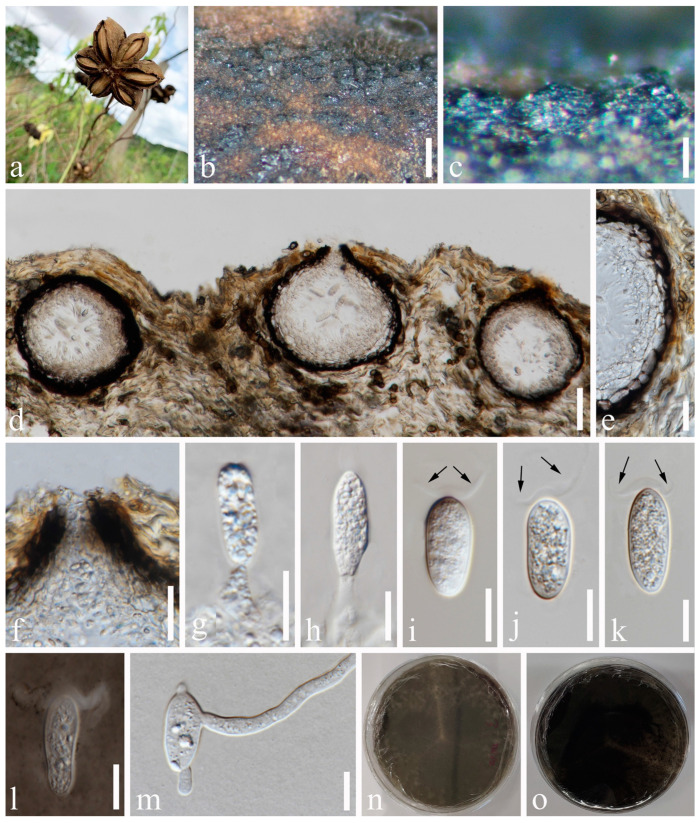
*Macrophomina euphorbiicola* (MFLU 23-0004). (**a**) Specimen. (**b**,**c**) Conidiomata on host substrate. (**d**) Vertical section of conidiomata. (**e**) Section of peridium. (**f**) Ostiole. (**g**,**h**) Conidiogenous cells and developing conidia. (**i**–**l**) Conidia bearing apical appendages (arrows). (**m**) Germinated conidium. (**n**,**o**) Colonies on PDA, above (**n**) and below (**o**). Scale bars: (**b**) = 50 μm, (**c**–**e**) = 20 μm, (**f**–**m**) = 10 μm.

*Macrophomina euphorbiicola* A.R. Machado, D.J. Soares & O.L. Pereira, Eur. J. Pl. Path. 153: 96 [43] Figure 12.

Index Fungorum number: IF815562.

*Saprobic* on dead seeds of *Plukenetia volubilis* L. Sexual morph: Not observed. Asexual morph: *Conidiomata* 116–172 × 130–161 μm ($\bar{x}$ = 137 × 149 μm, *n* = 20), circular, dark brown to black, solitary or gregarious, immersed through the epidermis, visible as black dots or papilla on the host, glabrous. *Peridium* up to 11–24 μm wide, composed of dark brown to black thick-walled *textura angularis*, becoming thin-walled and hyaline towards the inner region. *Ostiole* 12–32 μm diam., cylindrical, short, straight, centrally or laterally located. *Paraphyses* absent. *Conidiophores* subcylindrical to ampulliform, reduced to conidiogenous cells. *Conidiogenous cells* 6–14 × 2–5 μm ($\bar{x}$ = 9 × 3 μm, *n* = 20), terminal, hyaline, cylindrical to ellipsoidal, smooth-walled. *Conidia* 22–26 × 8–11 μm ($\bar{x}$ = 23 × 9 μm, *n* = 50), hyaline, oblong to cylindrical, with rounded apex, and narrow, straight, frequently constricted in the middle, aseptate, contents granular, thick- and smooth-walled, bearing octagonal beard-shaped appendages, or widely flared or irregular, undulate, mucoid apical appendage, basal appendages absent.

Culture characteristics: Conidia germinating on PDA within 12 h with germ tubes produced from the middle or each end. Colonies are fast growing on PDA, reaching 90 mm diam. after 5–6 days at 20–23 °C. Sparse, aerial, filamentous, after 2 weeks, becoming dark brown to black.

Material examined: Thailand, Chiang Rai Province, Thoeng, Tambon Nang Lae, Rai Ruen Rom Organic Farm, 19°39’30.2″ N, 100°09’26.4″ E, on dead seeds of *Plukenetia volubilis* L., 11 June 2019, Na Wu, YW62 (MFLU 23-0004), living culture MFLUCC 23-0057.

Notes: *Macrophomina euphorbiicola* was introduced by Machado et al. [43]. Due to the previous cultures failing to sporulate, comparison with the type species was not possible. The phylogenetic analysis showed that our isolate was nested within *M. euphorbiicola* and claded closer to *M. pseudophaseolina* (Figure 1). We, thus, identify the new collection as *M. euphorbiicola.*

**Figure 13:**
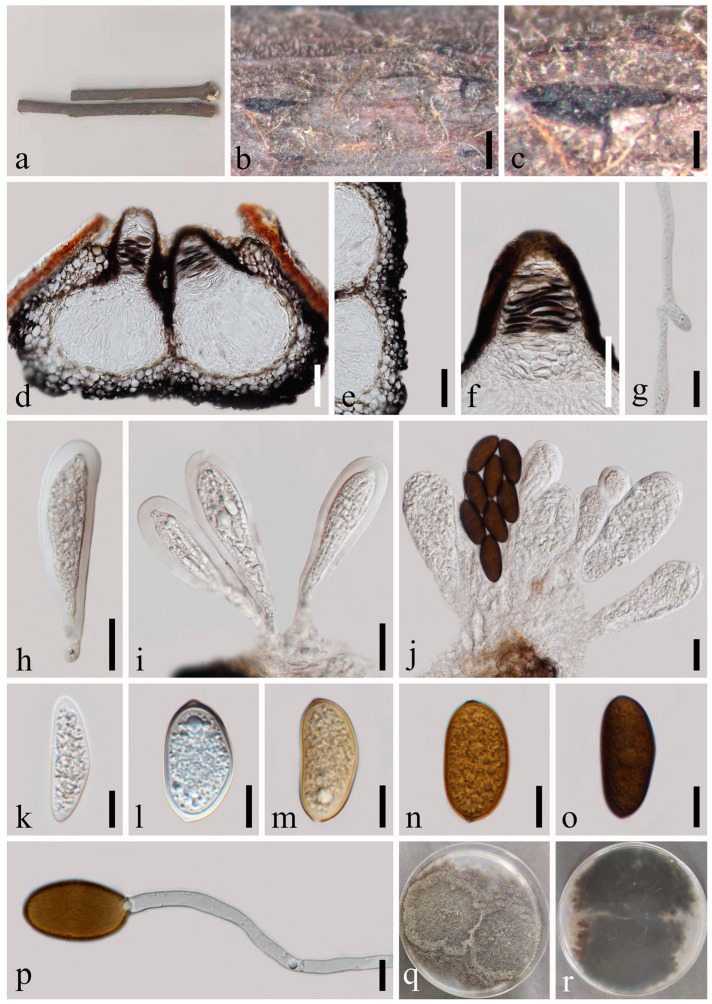
*Sphaeropsis eucalypticola* (MFLU 23-0008). (**a**–**c**) Ascomata on host substrate. (**d**) Vertical section of ascoma. (**e**) Structure of peridium. (**f**) Ostiole. (**g**) Pseudoparaphyses. (**h**–**j**) Asci. (**k**–**o**) Ascospores. (**p**) Germinated ascospore. (**q**,**r**) Colonies on PDA, above (**q**) and below (**r**). Scale bars: (**b**) = 50 μm, (**c**) = 20 μm, (**d**–**f**) = 50 μm, (**g**) = 10 μm, (**h**–**j**) = 20 μm, (**k**–**p**) = 10 μm.

*Sphaeropsis eucalypticola* A.J.L. Phillips, Stud. Mycol. 76: 158 [44] Figure 13.

Index Fungorum number: IF805464; Facesoffungi number: FoF00169.

*Saprobic* on dead twigs of *Tectona grandis*. Sexual morph: *Ascomata* 186–257 × 345–466 μm ($\bar{x}$ = 233 × 373 μm, *n* = 20), well-developed, bursting through the bark, generally strongly emerged from the host surface, scattered or gregarious, black, subglobose to obpyriform, ostiolate. *Ostiole* central, subconical to flattened and the region between the perithecial necks were occupied by black pseudoparenchymatous tissue. *Peridium* 31–59 μm wide, composed of three strata; an outer stratum is black, thick-walled cells, middle layer and inner layer, black thin-walled cells. *Hamathecium* comprising up to 2–5 μm wide, dense, cellular pseudoparaphyses, anastomosing above and between the asci. *Asci* 86–134 × 22–36 μm ($\bar{x}$ = 113 × 30 μm, *n* = 20), bitunicate, eight-spored, broadly clavate, apically rounded with a visible ocular chamber. *Ascospores* 29–34 × 15–19 μm ($\bar{x}$ = 32 × 17 μm, *n* = 50), initially rhomboid, hyaline, aseptate, becoming pigmented, brown to dark brown, elliptical to ovoid, thick-walled, smooth at maturity, with an apiculus at either end. Asexual morph: Not observed.

Culture characteristics: Ascospores germinating on PDA within 24 h. Colonies are fast growing on PDA, reaching 60 mm diam. after 5 d at 20–25 °C. Circular, white in first few days, becoming pale grey from the center after one week, and finally black after two weeks, felt-like, sparse, aerial, surface smooth with a crenate edge, filamentous.

Material examined: Thailand, Chiang Mai Province, Amphoe Mae Taeng, Tambon Cho Lae, 19°08’01.3″ N, 99°00’29.4″ E, on dead twigs of *Tectona grandis* (*Lamiaceae*), 6 August 2019, Na Wu, YW174 (MFLU 23-0008), living culture MFLUCC 23-0040; *ibid*., 7 August 2019, Na Wu, YW213 (GZAAS 23-0589), living culture GZCC 23-0589.

Notes: The phylogenetic results (Figure 1) showed that our newly obtained isolate clustered together with *Sphaeropsis eucalypticola*, and we identified it as *S. eucalypticola*. This is the first record of *S. eucalypticola* found on *Tectona grandis* in Thailand.

## 4. Discussion

Studies on *Botryosphaeriaceae*, dealing with the phylogenetic traits and morphology of isolates associated with various hosts, have increased in recent years, enabling the worldwide identification of taxa at the species level [2,5,18,45,46,47,48,49]. In this study, 16 *Botryosphaeriaceae* isolates were obtained from several decaying woody hosts (dead arial twigs, branches, stems, bark, and seed pods) in southwestern China and northern Thailand, and they were identified as 11 species based on a polyphasic approach of morphological features and molecular phylogeny. These species included *Botryosphaeria fujianensis*, *Diplodia mutila*, *Di. seriata*, *Dothiorella ovata*, *Do. rosacearum*, *Do. septata*, *Lasiodiplodia crassispora*, *L. delonicis*, *L. mahajangana*, *Macrophomina euphorbiicola* and *Sphaeropsis eucalypticola.* Of these, *Do. ovata*, *Do. rosacearum*, *Do. septata* and *L. delonicis* are introduced as novel species, and the remaining seven species were identified as new hosts or new geographical records. All species collected in this study are saprophytic on the host. It should be noted that even though sporulation was induced on sterile toothpicks or pine needles on PDA, the respective asexual morph or sexual morph was not observed. Thus, the fungal identification and classification in this study are based on their morphological characteristics of either asexual or sexual morphs and the phylogenetic analysis results.

*Macrophomina* and *Sphaeropsis* are two of the least common genera in the family *Botryosphaeriaceae*. Five species are validly known in *Macrophomina*, among which *M. phaseolina* and *M. euphorbiicola* were introduced as pathogens [43,50,51,52]. In this study, the asexual morph of *M. euphorbiicola* was collected from *Plukenetia volubilis* in northern Thailand. Due to the previous cultures failing to sporulate, the morphology of *M. euphorbiicola* has not been described [43]. Hence, we provide the first detailed description and illustration of *M. euphorbiicola* for the first time and also report it as a new record from *Plukenetia volubilis* in Thailand. *Sphaeropsis* was typified with *S. visci* by Saccardo [53] with 632 records in Index Fungorum (Accessed July 2023), and only eight species are recognized with accessible cultures so far [16,44]. In this study, one previously known species, *S. eucalypticola*, was collected from *Tectona grandis* in Thailand and reported as a new host record. *Sphaeropsis eucalypticola* has also been reported on *Bauhinia purpurea* and *Eucalyptus* sp. in Thailand [4,54]. Mapook et al. [55] identified *S. chromolaenicola* from *Chromolaena odorata* in Thailand. However, the remaining members of the genus have not been found on any host in Thailand.

This study revealed two previously known *Diplodia* species, *Di. mutila* and *Di. seriata* from Sichuan province. It is worth noting that similarly to our collection of *Di. mutila* (from *Prunus persica*) and *Di. seriata* (from *Wisteria sinensis*), Li et al. [49] also found these two species from dead branches of *Camellia oleifera*, and another two *Diplodia* species (*Di. acerigena* and *Di. pistaciicola*) in Sichuan province. *Diplodia* species mainly occur on woody hosts, causing rots, cankers, shoot and tip blight [11,56,57,58,59]. Thus, the discovery and in-depth research of this genus are conducive to the protection of woody plants and the maintenance of greater economic benefits.

*Dothiorella* was the most frequently isolated genus in this study, as seven *Dothiorella* isolates were obtained from decaying woody hosts in Chiang Mai Province, Thailand. *Dothiorella* was introduced by Saccardo [53] with *Do. pyrenophora* as the type species, and presently, only 38 species are accepted in this genus based on phylogenetic analyses [16,18,49]. Zhang et al. [48] made a systematic revision of *Dothiorella* by synonymizing 15 known species, which reduced the number of *Dothiorella* members and established a more stable systematic relationship. Most of the members of the genus *Dothiorella* were rarely collected in Thailand in the past; however, there have been many reports of *Dothiorella* species being collected in Thailand in recent years [60,61,62,63]. We speculate that this may be due to random sampling. In this study, three new species *Do. ovata*, *Do. rosacearum* and *Do. septata* are introduced based on morphological features (asexual morphs) and phylogenetic evidence.

*Lasiodiplodia* was formally established by Clendenin [64] with *L. tubericola* Ellis and Everhart (=*L. theobromae*) [4] as the type species. So far, 37 ex-type/isotype/neotype species entries have been accepted and uploaded to the *Botryosphaeriales* website [16,65,66]. It is worth noting that most of the species were introduced as asexual morphs of *Lasiodiplodia*, and only a few species of sexual morph have been found in nature, such as *L. gonubiensis*, *L. lignicola* and *L. theobromae* [44,67,68]. The three *Lasiodiplodia* species collected in this study were all asexual morphs and collected from woody plants. Among them, *L. crassispora* and *L. mahajangana* were previously known species, and *L. delonicis* was introduced as a new species. *Lasiodiplodia crassispora* was first introduced by Burgess et al. [41] based on distinctive morphological characters and phylogenetic analyses. Zhang et al. [48] synonymized *L. pyri* under *L. crassispora.* In this study, *L. crassispora* was collected from decaying wood. Though the species has been found in several countries, such as Australia, Brazil, Namibia, Senegal, and Venezuela [41,48], this is the first time *L. crassispora* has been reported in Thailand. We collected *L. mahajangana* from *Dipterocarpus retusus* in this study. Zhang et al. [48] synonymized *L. caatinguensis*, *L. curvata*, *L. exigua*, *L. irregularis*, *L. macroconidia*, and *L. pandanicola* under *L. mahajangana*, thus expanding the host range and geographical distribution of this species. Interestingly, the conidia of *L. mahajangana* are straight or curved, and its conidia morphology is more special compared with other species of *Lasiodiplodia* [42,48]. At the same time, this study also collected a new species, *L. delonicis*, from a fallen pod of *Delonix regia*. In addition, mature conidia with longitudinal striations of *Lasiodiplodia* is one of its distinguishing features from *Diplodia* [44]. However, it has been observed that if the asexual stage is produced on culture, the conidia often have obvious longitudinal striations, while the asexual stage produced in nature has less distinct or absent longitudinal striations [61,68,69,70]. This inference can be found in previous reports and this study. The reason for this phenomenon might be due to the variations in the environment in which the fungi grow. Thus, it is important to collect more fresh specimens to verify this observation.

*Botryosphaeria fujianensis* was introduced as a pathogen-causing stem canker of blueberry in Fujian province, China [37], whereas our species was isolated from dead twigs of *Tectona grandis* (*Lamiaceae*) in Chiang Mai Province, Thailand. As this is the first record of *B. fujianensis* isolated from Thailand, we suspect that it might be found on more hosts in the future.

With the increased number of studies of *Botryosphaeriaceae* based on morphology, ecology, and DNA-based phylogeny, more new species and records are constantly being discovered [7,71,72,73,74]. However, there are still many aspects needed to clarify this fungal family, such as specifying species from environmental samples, resolving the opportunistic pathogenic nature, and defining species boundaries. The results of this study indicate that there is still much potential for *Botryosphaeriaceae* members to be discovered in China and Thailand. As members of the *Botryosphaeriaceae* family represent a growing threat to agricultural crops and urban and natural forest ecosystems [75,76,77,78], this finding raises questions about the origin, introduction, and pathway of these fungi as well as underlining the need to develop suitable actions to limit their further spread.

## Figures and Tables

**Table 1:** Primers and PCR protocols used in this study.

Locus	Primers	Optimized PCR Protocols	Reference
ITS	ITS5	94 °C 3 min; 35 cycles of 94 °C 30 s, 55 °C 50 s, 72 °C 1 min; 72 °C 10 min; 4 °C on hold	[25]
	ITS4		
LSU	LR0R	94 °C 3 min; 35 cycles of 94 °C 30 s, 55 °C 50 s, 72 °C 1 min; 72 °C 10 min; 4 °C on hold	[26]
	LR5		
*tef1-α*	EF1-728F	94 °C 3 min; 35 cycles of 94 °C 30 s, 55 °C 50 s, 72 °C 1 min; 72 °C 10 min; 4 °C on hold	[27]
	EF1-986R		
*tub2*	Bt2a	94 °C 3 min; 35 cycles of 94 °C 30 s, 55 °C 50 s, 72 °C 1 min; 72 °C 10 min; 4 °C on hold	[28]
	Bt2b		

**Table 2:** Taxa Names, Strain or Specimen numbers, and corresponding GenBank accession numbers of the taxa used for the phylogenetic studies. The newly generated sequences are indicated in red, and ex-type strains are indicated in bold.

Taxa Names	Strain/Specimen Numbers	GenBank Accession Numbers			
		ITS	LSU	*tef1-α*	*tub2*
***Alanphillipsia aloeicola***	CBS 138896	KP004444	KP004472	MT592027	–
*Alanphillipsia aloetica*	CBS 136409	KF777139	KF777195	MT592028	–
***Barriopsis archontophoenicis***	MFLUCC 14-1164	KX235306	KX235307	KX235305	–
***Barriopsis stevensiana***	CBS 174.26	EU673330	DQ377857	EU673296	–
***Botryobambusa fusicoccum***	MFLUCC 11-0143	JX646792	JX646809	JX646857	–
***Botryobambusa guizhouensis***	CGMCC 3.20348	MZ781425	MZ781492	MZ852498	–
***Botryosphaeria dothidea***	CBS 115476	AY236949	AY928047	AY236898	AY236927
***Botryosphaeria dolichospermatii***	CGMCC 3.19096	MH491970	MH562323	MH491974	MH562327
***Botryosphaeria fabicerciana***	CBS 127193	HQ332197	MF410028	HQ332213	KF779068
*Botryosphaeria fabicerciana*	CBS 127194	HQ332198	MF410029	HQ332214	KF779069
***Botryosphaeria fujianensis***	CGMCC 3.19099	MH491973	MH562326	MH491977	MH562330
*Botryosphaeria fujianensis*	BJFUCC 180226-3	MW251380	MW251381	MW251388	MW251379
*Botryosphaeria fujianensis*	MFLUCC 23 - 0041	OR052056	OR052040	OR030453	OR030471
***Botryosphaeria qingyuanensis***	CGMCC 3.18742	KX278000	MF410042	KX278105	KX278209
***Botryosphaeria tenuispora***	MUCC 237	LC585278	–	LC585150	LC585174
***Cophinforma eucalypti***	MFLUCC 11-0425	JX646800	JX646817	JX646865	JX646848
*Cophinforma eucalypti*	MFLUCC 11-0655	JX646801	JX646818	JX646866	JX646849
***Cophinforma mamane***	CBS 117444	KF531822	DQ377855	KF531801	KF531802
*Diplodia mutila*	CBS 112553	AY259093	AY928049	AY573219	DQ458850
*Diplodia mutila*	CBS 112875	AY343484	–	AY343370	MT592509
*Diplodia mutila*	GZCC 23 - 0578	OR052057	OR020607	OR030454	OR030472
***Diplodia neojuniperi***	CBS 138652	KM006431	–	KM006462	MT592516
***Diplodia sapinea***	CBS 393.84	DQ458895	DQ377893	DQ458880	DQ458863
***Diplodia scrobiculata***	CBS 118110	AY253292	KF766326	AY624253	AY624258
***Diplodia seriata***	CBS 112555	AY259094	AY928050	AY573220	DQ458856
*Diplodia seriata*	CBS 112661	MT587378	–	MT592084	MT592541
*Diplodia seriata*	GZCC 23 - 0579	OR052058	OR052041	OR030455	OR030473
***Diplodia subglobosa***	CBS 124133	GQ923856	–	GQ923824	MT592576
***Dothiorella acacicola***	CBS 141295	KX228269	KX228320	KX228376	–
***Dothiorella acericola***	KUMCC 18-0137	MK359449	–	MK361182	–
***Dothiorella albiziae***	MFLUCC 22-0057	ON751762	ON751764	ON799588	ON799590
***Dothiorella alpina***	CGMCC 3.18001	KX499645	–	KX499651	–
***Dothiorella baihuashanensis***	CFCC 58549	–	–	OQ692933	OQ692927
*Dothiorella baihuashanensis*	CFCC 58788	–	–	OQ692934	OQ692928
***Dothiorella brevicollis***	CBS 130411	JQ239403	JQ239416	JQ239390	JQ239371
***Dothiorella camelliae***	CGMCC 3.24158	OQ190531	–	OQ241464	OQ275064
***Dothiorella capri-amissi***	CBS 121763	EU101323	KX464301	EU101368	KX464850
***Dothiorella casuarinae***	CBS 120688	DQ846773	MH874647	DQ875331	DQ875340
*Dothiorella citricola*	CBS 124728	EU673322	–	EU673289	KX464852
***Dothiorella citrimurcotticola***	CGMCC 3.20394	MW880661	–	MW884164	MW884193
*Dothiorella citrimurcotticola*	CGMCC 3.20395	MW880662	–	MW884165	MW884194
***Dothiorella diospyricola***	CBS 145972	MT587398	–	MT592110	MT592581
***Dothiorella dulcispinae***	CBS 130413	JQ239400	JQ239413	JQ239387	JQ239373
*Dothiorella dulcispinae*	CMW 36462	JQ239402	JQ239415	JQ239389	JQ239375
***Dothiorella eriobotryae***	CBS 140852	KT240287	–	KT240262	MT592582
***Dothiorella heterophyllae***	CMW46458	MN103794	–	MH548348	MH548324
***Dothiorella iranica***	CBS 124722	KC898231	–	KC898214	KX464856
***Dothiorella koae***	CMW 48017	MH447652	–	MH548338	MH548327
***Dothiorella lampangensis***	MFLUCC 18-0232	MK347758	–	MK340869	MK412874
***Dothiorella longicollis***	CBS 122068	EU144054	MH874718	EU144069	KF766130
***Dothiorella magnoliae***	CFCC 51563	KY111247	–	KY213686	–
***Dothiorella mangifericola***	IRAN 1584C	MT587407	–	MT592119	–
***Dothiorella moneti***	MUCC 505	EF591920	EF591937	EF591971	EF591954
***Dothiorella obovata***	MFLUCC 22-0058	ON751763	ON751765	ON799589	ON799591
***Dothiorella ovata***	MFLUCC 23 - 0035	OR052059	OR020691	OR030456	OR030474
*Dothiorella ovata*	MFLUCC 23 - 0036	OR052060	OR052042	OR030457	OR030475
***Dothiorella plurivora***	CBS 124724	KC898225	–	KC898208	KX464874
***Dothiorella pretoriensis***	CBS 130404	JQ239405	JQ239418	JQ239392	JQ239376
***Dothiorella prunicola***	CBS 124723	EU673313	EU673232	EU673280	EU673100
***Dothiorella rosacearum***	MFLUCC 23 - 0038	OR052061	OR052043	OR030458	OR030476
*Dothiorella rosacearum*	MFLUCC 23 - 0037	OR052062	OR052044	OR030459	OR030477
***Dothiorella santali***	WAC 13155	EF591924	EF591941	EF591975	EF591958
***Dothiorella sarmentorum***	CBS 115038	AY573206	DQ377860	AY573223	EU673101
*Dothiorella sarmentorum*	IMI 63581b	AY573212	AY928052	AY573235	–
***Dothiorella septata***	MFLUCC 23 - 0039	OR020942	OR020695	OR030462	OR030480
*Dothiorella septata*	GZCC 23 - 0583	OR019776	OR052047	OR030463	OR030481
*Dothiorella septata*	GZCC 23 - 0584	OR019803	OR052048	OR030464	OR030482
***Dothiorella striata***	CBS 124731	EU673321	–	EU673288	EU673143
*Dothiorella striata*	CBS 124730	EU673320	EU673240	EU673287	EU673142
***Dothiorella tectonae***	MFLUCC 18-0382	KM396899	–	KM409637	KM510357
***Dothiorella thailandica***	MFLUCC 11-0438	JX646796	JX646813	JX646861	JX646844
***Dothiorella thripsita***	CBS 125445	FJ824738	–	KJ573639	KJ577550
***Dothiorella ulmacea***	CBS 138855	KR611881	KR611899	KR611910	KR611909
*Dothiorella ulmacea*	CBS 140005	KR611882	–	KR857697	MT592607
***Dothiorella uruguayensis***	CBS 124908	EU080923	MH874932	EU863180	KX464886
***Dothiorella vinea-gemmae***	DAR 81012	KJ573644	–	KJ573641	KJ577552
***Dothiorella viticola***	CBS 117009	AY905554	MH874565	AY905559	EU673104
*Dothiorella yunnana*	CGMCC 3.18000	KX499644	–	KX499650	–
*Dothiorella zanthoxyli*	CGMCC 3.24159	OQ190536	–	OQ241468	OQ275069
***Endomelanconiopsis endophytica***	CBS 120397	EU683656	EU683629	EU683637	KF766131
***Endomelanconiopsis freycinetiae***	MFLUCC 17-0547	MG646955	MG646948	MG646983	MG646924
*Eutiarosporella africana*	CMW 38423	KC769956	KC769990	KC769852	–
***Eutiarosporella dactylidis***	MFLUCC 13-0276	KM978944	KM978949	KP031694	–
*Eutiarosporella urbis-rosarum*	CMW 36477	JQ239407	JQ239420	JQ239394	JQ239381
***Lasiodiplodia crassispora***	CBS 118741	DQ103550	DQ377901	DQ103557	KU887506
*Lasiodiplodia crassispora*	CMW 13488	DQ103552	–	DQ103559	KU887507
*Lasiodiplodia crassispora*	MFLUCC 23-0060	OR052065	OR020699	OR030465	OR030483
***Lasiodiplodia delonicis***	MFLUCC 23-0058	OR052066	OR052049	OR030466	OR030484
***Lasiodiplodia mahajangana***	CBS 124925	FJ900595	–	FJ900641	FJ900630
*Lasiodiplodia mahajangana*	CBS 124926	FJ900596	–	FJ900642	FJ900631
*Lasiodiplodia mahajangana*	MFLUCC 23-0059	OR052067	OR052050	OR030467	OR030485
***Lasiodiplodia rubropurpurea***	WAC 12535	DQ103553	DQ377903	DQ103571	EU673136
*Lasiodiplodia rubropurpurea*	WAC 12536	DQ103554	–	DQ103572	KU887530
***Lasiodiplodia thailandica***	CBS 138760	KJ193637	–	KJ193681	–
*Lasiodiplodia thailandica*	CBS 138653	KM006433	–	KM006464	–
***Lasiodiplodia theobromae***	CBS 164.96	AY640255	EU673253	AY640258	KU887532
*Lasiodiplodia theobromae*	CBS 111530	EF622074	–	EF622054	KU887531
***Lasiodiplodia venezuelensis***	WAC 12539	DQ103547	DQ377904	DQ103568	KU887533
*Lasiodiplodia venezuelensis*	WAC 12540	DQ103548	–	DQ103569	KU887534
***Lasiodiplodia viticola***	CBS 128313	HQ288227	KX098286	HQ288269	HQ288306
*Lasiodiplodia viticola*	CBS 128314	HQ288228	–	HQ288270	HQ288307
***Macrophomina euphorbiicola***	CMM 4134	KU058936	–	KU058906	MF457658
*Macrophomina euphorbiicola*	CMM 4045	KU058928	–	KU058898	MF457657
*Macrophomina euphorbiicola*	MFLUCC 23-0057	OR052068	OR052051	OR030468	OR030486
***Macrophomina phaseolina***	CBS 205.47	KF951622	–	KF951997	MW592323
***Macrophomina pseudophaseolina***	CBS 137165	KF951791	–	KF952153	KF952233
***Macrophomina tecta***	BRIP 70781	MW591684	–	MW592271	MW592300
***Marasasiomyces karoo***	CBS 118718	KF531828	DQ377939	KF531807	KF531808
***Mucoharknessia cortaderiae***	CPC 19974	KM108374	KM108401	–	–
***Mucoharknessia anthoxanthii***	MFLUCC 15-0904	KU246377	KU246379	–	–
***Neodeightonia subglobosa***	CBS 448.91	EU673337	DQ377866	EU673306	EU673137
***Neodeightonia phoenicum***	CBS 122528	EU673340	EU673261	EU673309	EU673116
*Neodeightonia phoenicum*	CBS 123168	EU673339	EU673260	EU673308	EU673115
*Neoscytalidium dimidiatum*	CBS 145.78	KF531816	DQ377922	KF531795	KF531796
*Neoscytalidium dimidiatum*	CBS 251.49	KF531819	DQ377923	KF531797	KF531799
***Oblongocollomyces variabilis***	CBS 121774	EU101312	KX464536	EU101357	–
*Oblongocollomyces variabilis*	CBS 121775	EU101314	MT587319	EU101359	–
***Phaeobotryon mamane***	CBS 122980	EU673332	EU673248	EU673298	–
***Phaeobotryon cupressi***	CBS 124700	FJ919672	KX464538	FJ919661	–
***Sakireeta madreeya***	CBS 532.76	KC769960	DQ377940	KM108427	KX465084
***Sardiniella celtidis***	MFLUCC 17-981	MF443249	–	MF443248	–
***Sardiniella urbana***	CBS 141580	KX379674	KX379676	KX379675	–
*Sardiniella urbana*	BL180	KX379677	KX379679	KX379678	–
***Sphaeropsis citrigena***	ICMP 16812	EU673328	EU673246	EU673294	EU673140
***Sphaeropsis eucalypticola***	MFLUCC 11-0579	JX646802	JX646819	JX646867	JX646850
*Sphaeropsis eucalypticola*	MFLUCC 11-0654	JX646803	JX646820	JX646868	JX646851
*Sphaeropsis eucalypticola*	MFLUCC 23 - 0040	OR052069	OR052052	OR030469	OR030488
*Sphaeropsis eucalypticola*	GZCC 23 - 0589	OR052070	OR052053	OR030470	OR030487
***Sphaeropsis porosa***	CBS 110496	AY343379	DQ377894	AY343340	EU673130
*Sphaeropsis visci*	CBS 100163	EU673324	EU754215	EU673292	EU673127
***Sphaeropsis guizhouensis***	CGMCC 3.20352	MZ781433	MZ781500	MZ852506	–
***Tiarosporella palludosa***	CPC 22701	KM108378	KM108404	–	KM108471
*Tiarosporella palludosa*	CPC 22702	KM108379	KM108405	–	KM108472
*Neofusicoccum mangiferae*	CBS 118531	AY615185	DQ377921	DQ093221	AY615172
***Neofusicoccum parvum***	CMW 9081	AY236943	AY928045	AY236888	AY236917
*Neofusicoccum parvum*	CBS 110301	AY259098	AY928046	AY573221	EU673095
***Pseudofusicoccum adansoniae***	CBS 122055	EF585523	–	EF585571	MT592771
*Pseudofusicoccum adansoniae*	CBS 122056	EF585524	–	MT592279	MT592772

## Data Availability

The datasets generated for this study can be found in the NCBI database.
